# Editorial: Innovative microbial technologies for future and sustainable food science

**DOI:** 10.3389/fmicb.2023.1215775

**Published:** 2023-06-12

**Authors:** Yu Xia, Zhe Zeng, Ana López Contreras, Chun Cui

**Affiliations:** ^1^State Key Laboratory of Food Science and Technology, School of Food Science and Technology, Jiangnan University, Wuxi, China; ^2^Wageningen University and Research, Wageningen, Netherlands; ^3^School of Food Science and Engineering, South China University of Technology, Guangzhou, China

**Keywords:** food microbiology, microbial technology, future foods, sustainability, food safety and control, food processing and brewing

## Introduction

Microbial technology has revolutionized the field of food sciences, allowing for the development of new food products and food ingredients, improving food safety and even better understanding on the interactions in the gut microbiome. Microorganisms play a crucial role in production of many foods, including cheese, yogurt, fermented meats, and alcoholic beverages. Advances in microbial technology have made it possible to better understand and manipulate these microorganisms, leading to current and future innovations. The current Research Topic collects both original research articles and reviews on traditional food fermentation, i.e. Chinese liquor fermentation (Hou et al.; Shi et al.) and yogurt production (El-Sayed et al.; Xiao et al.), sustainable production of food ingredients, e.g., isomaltulose (Hu et al.), D-allulose (Chen et al.) and ε-poly-L-lysine (Liu et al.), food safety (Wu et al.) and explorative innovation on gut microbiome (Chen et al.; Gao et al.; Yue et al.).

## Beyond traditional food fermentation

Traditional food fermentation is a process that has been used for centuries to preserve and transform foods through the activity of microorganisms. Among the most widely known traditional fermentation practices are Chinese liquor fermentation and yogurt production. One of the most significant advances in microbial technology has been the development of starter cultures. In this topic, Shi et al. presented a study on the yeast *Wickerhamomyces anomalus* Y-1, which is commonly found in Chinese liquor fermentation starters, and the researchers used genomic and transcriptomic analysis to identify genes involved in flavor metabolism in this yeast and found that it produces a range of volatile compounds that contribute to the aroma and flavor of Chinese liquor. Hou et al. sheds light on the dynamic changes of Daqu, a type of fermentation starter used in Chinese liquor production, and provides insight into the factors that influence microbial communities and physicochemical properties during fermentation. Xiao et al. unveiled properties and nutritional composition of kefir yogurt, which is a fermented dairy product resulting from adding kefir grains to milk, while El-Sayed et al. showed that stirred yogurt as a matrix for freeze-dried microcapsules could be an effective and tasty way to deliver synbiotic compounds to consumers. In short, microbial technology has significant implications for traditional food fermentation, contributing to controlling the fermentation process and the production of safe, tasty, and nutritious foods.

## Sustainable production of food ingredient

Microbial synthesis of food using synthetic biology is now recognized as a sustainable and efficient approach for scaling up food production (Lv et al., 2021; Arun et al., 2023). Synthetic biology enables the design and construction of novel biomolecular components, pathways, and networks that can reprogram organisms to serve as engineered cell factories (Khalil and Collins, 2010; Lv et al., 2021). In this Research Topic, Hu et al. described a sustainable approach to produce isomaltulose, a non-cariogenic, slow-release carbohydrate, using an engineered food-grade strain of *Corynebacterium glutamicum*. By improving thermostability of sucrose isomerase and using one-step simplified cell immobilization, the production of isomaltulose was significantly increased up to 453.0 g/L when 500.0 g/L sucrose solution was used as substrate with reduced environmental impact. Liu et al. applied genome shuffling on strains obtained by ribosome engineering to generate a better ε-PL producing strain from parental strain *Streptomyces albulus* M-Z1Z8. Chen et al. systematically reviewed recent advances in the physiological functions and biosynthesis of D-allulose, a rare sugar with potential health benefits, emphasizing on the enzymes and metabolic pathways involved in D-allulose biosynthesis, highlighting the potential of microbial engineering to improve production efficiency. Application of synthetic biology in sustainable production of food ingredients will expand the range of available feedstocks and potentially lead to more sustainable and high-quality food production.

## Innovation on gut microbiome

Today, more and more evidence has proved that gut microbiome plays a crucial role in our bodies, influencing our immune systems, metabolisms, and even our moods and behaviors (Gilbert et al., 2018). Innovative microbiome-related applications have created a gigantic market ranging from functional foods (e.g., foods enriched in prebiotic fibers and/or probiotic bacteria) to dietary supplements (e.g., probiotic capsules or powders) and therapeutic applications (e.g., gut microbiota transplant) (D'Hondt et al., 2021). Gao et al. investigated the effects of gut microbiota transplantation (GMT) from high-fat-diet-tolerant cynomolgus monkeys on hyperlipidemia and hepatic steatosis in rats. The results showed that the GMT led to a significant reduction in serum lipid levels and hepatic lipid accumulation in the recipient rat, suggesting that GMT may be a potential therapeutic strategy for treating metabolic disorders, such as hyperlipidemia and hepatic steatosis. Chen et al. investigated the differences in the gastric juice microbiota of pediatric patients with chronic gastritis who tested positive or negative for *Helicobacter pylori*. This study suggested that the gastric juice microbiota may play a role in the pathogenesis of chronic gastritis and could be a potential target for future therapies. On-going research of the microbiome has created an opportunity for the bioeconomy approach, providing practical solutions with potential for commercial application and enhancing social wellbeing.

## Author contributions

ZZ and YX prepared the manuscript. YX revised the contexts. AL and CC provided comments to the manuscript. All authors approved the submission of this manuscript.
